# Repeated Irradiation with γ-Ray Induces Cancer Stemness through TGF-β-DLX2 Signaling in the A549 Human Lung Cancer Cell Line

**DOI:** 10.3390/ijms22084284

**Published:** 2021-04-20

**Authors:** Hae-Ran Park, Yeo-Jin Choi, Jee-Young Kim, In-Gyu Kim, Uhee Jung

**Affiliations:** 1 Biotechnology Research Division, Korea Atomic Energy Research Institute, Jeonbuk 56212, Korea; yjchoi@kaeri.re.kr (Y.-J.C.); jykim@kaeri.re.kr (J.-Y.K.); 2 Environmental Safety Evaluation Research Division, Korea Atomic Energy Research Institute, Daejeon 34057, Korea; igkim@kaeri.re.kr

**Keywords:** ionizing radiation, DLX2, non-small cell lung cancer, radioresistance, cancer stem cell, epithelial to mesenchymal transition (EMT)

## Abstract

Cancer stem cells (CSCs) play an important role in cancer recurrence and metastasis. It is suggested that the CSC properties in heterogeneous cancer cells can be induced by ionizing radiation (IR). This study investigated the role of DLX2 in the radioresistance and CSC properties induced by IR in NSCLC cancer cells. Here, A549 cells were exposed to fractionated irradiation at a cumulative dose of 52 Gy (4 Gy × 13 times) for a generation of radioresistant cells. After fractionated irradiation, surviving A549 cells exhibited resistance to IR and enhanced expression of various cancer stem cell markers. They also showed upregulation of mesenchymal molecular markers and downregulation of epithelial molecular markers, correlating with an increase in the migration and invasion. Fractionated irradiation triggered the secretion of TGF-β1 and DLX2 expression. Interestingly, the increased DLX2 following fractionated irradiation seemed to induce the expression of the gene for the EGFR-ligand betacellulin via Smad2/3 signaling. To contrast, DLX2 knockdown dramatically decreased the expression of CSC markers, migration, and proliferation. Moreover, A549 cells expressing DLX2 shRNA formed tumors with a significantly smaller volume compared to those expressing control shDNA in a mouse xenograft assay. These results suggest that DLX2 overexpression in surviving NSCLC cancer cells after fractionated IR exposure is involved in the cancer stemness, radioresistance, EMT, tumor survival, and tumorigenic capability.

## 1. Introduction

Ionizing radiation (IR) is one of the most common and effective tools for cancer therapy. During radiotherapy for cancer, repeated and fractionated irradiation at a low dose reduces adverse effects, and allows damaged normal cells to recover [1]. However, several studies have suggested that this conventional radiotherapy eventually can lead to tumor recurrence and metastasis [2,3,4]. According to several recent studies, cancer stem cells (CSCs) are considered responsible for tumor relapse, metastasis, and resistance to IR and anti-cancer drugs [5,6,7,8,9]. CSCs have been isolated from various human malignant cancers [10,11,12,13,14,15], although the exact origin of CSCs remains debated. Representative CSC markers have been suggested, such as Oct4, Sox2, Snail, CD44, CD133, CD166, and aldehyde dehydrogenase (ALDH) in human cancer [16,17,18,19,20,21,22].

It has been reported that IR can induce stem cell-related phenotypes in heterogenous cancer cells and epithelial to mesenchymal transition (EMT) in vitro [23,24,25,26,27]. Cancer cells that undergo EMT are characterized by the losses of epithelial morphology, E-cadherin, and other components of epithelial cell junctions. Instead, they produce a mesenchymal cell cytoskeleton such as N-cadherin, vimentin, β-catenin, and fibronectin. Through these series of EMT processes, cancer cells acquire the properties of migration and invasion, which can be the initiation of metastasis, and also achieve stemness phenotypes and radioresistance [28,29,30]. EMT is activated via several signaling pathways including TGF-β, Wnt, and Notch, and is regulated by transcription factors such Twist, Slug, and Snail [31,32,33]. 

Occurring at the early stages of tumorigenesis, TGF-β acts as a tumor suppressor by promoting growth arrest and apoptosis [34,35]. However, TGF-β also is known to promote tumor progression by enhancing EMT and angiogenesis [35,36,37]. Tumor-suppressive TGF-β signaling is mediated by canonical, Smad-dependent TGF-β signaling, but canonical signaling in cancer cells is often suppressed. To contrast, non-canonical TGF-β signaling promotes tumor progression and survival, which mainly results in the activation of MAPK and PI3K pathways. Several reports showed that IR activates Smad-dependent TGF-β signaling, leading to promotion of EMT, migration, and invasion in cancer cells. Additionally, it was previously reported that single irradiation in A549 and MDA-MB-231 cell lines induced the stem cell-like properties and EMT by activating Smad2/3 signaling, which results in the upregulation of DLX2 [38]. 

Distal-less homeobox-2 (DLX2) is one of the vertebrate distal-less gene family proteins that play a crucial role in regulating embryonic development and tissue homeostasis [39,40]. Recently, increased DLX2 expression has been found in various solid tumors and hematologic malignancies [41,42,43]. DLX2 acts as an upstream regulator of Snail, which is involved in TGF-β and Wnt-induced EMT, oncogenic metabolism, and mitochondrial repression [44]. Interestingly, DLX2 is induced by TGF-β, leading to the shift of TGF-β action from tumor suppression into tumor promotion [42]. Although these previous studies showed the potential role of DLX2 in the tumor progression and metastasis involving TGF-β signaling, the detailed biological role of DLX2 in the acquirement of CSC and EMT characteristics of irradiated cancer cells remains largely unknown. 

During this study, we showed that repeated IR induced cancer stemness properties and EMT with the upregulation of TGF-β and DLX2 signaling in the A549 human lung cancer cell line. Moreover, we showed that DLX2 can suppress TGF-β signaling as well as CSC and EMT characteristics and growth of A549 cells.

## 2. Results

### 2.1. Surviving A549 Cells after Fractionated Irradiation Acquire Radioresistance and Stemness 

**Figure 1:**
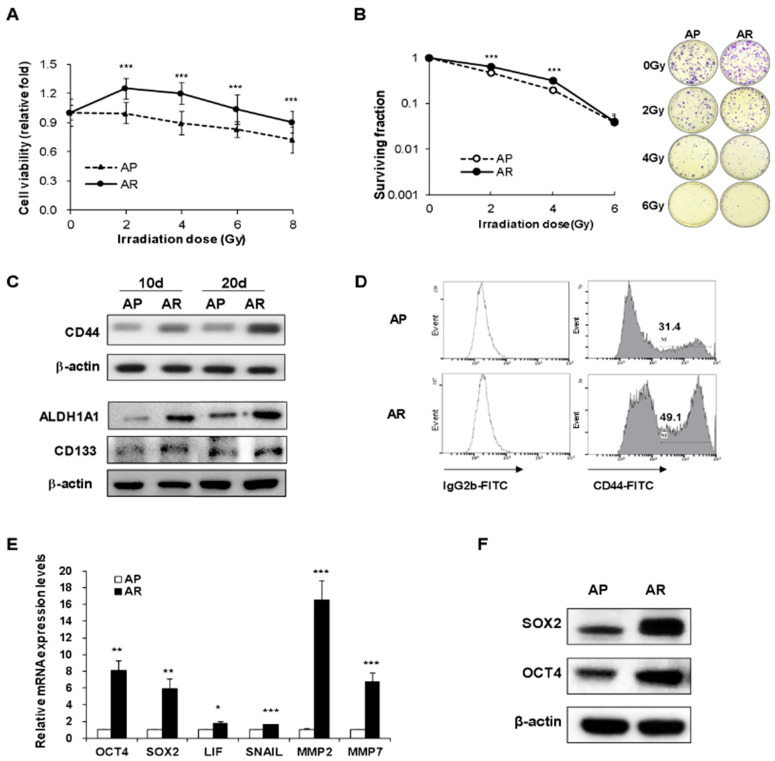
Fractionated ionizing radiation promotes the radioresistance and the expression of cancer stemness-related markers in A549 cells. (**A**) Both AP cells and AR cells were irradiated at 2 Gy, 4 Gy, 6 Gy or 8 Gy. After irradiation, both cells (1 × 10^4^/well) were cultured for 24 h and then the cell viability was determined by CCK-8. The bars indicate the mean of ten wells from two independent experiments. (**B**) Both AP cells and AR cells were irradiated at 2 Gy, 4 Gy or 6 Gy. Seven days after irradiation, colonies containing 50 cells or more were counted as clonogenic cells and the results were expressed as a surviving fraction. The bars indicate the mean of eighteen wells from three independent experiments. (**C**) The expression levels of CD44, ALDH1A1, and CD133 proteins in AP and AR cells were measured by Western blot analysis. (**D**) The expression of CD44 in AP and AR cells was analyzed by flow cytometry. (**E**) The mRNA expression levels of stemness markers (OCT4, SOX2, LIF), EMT-related transcription factor (Snail), and metastasis markers (MMP2, MMP7) in both AP and AR cells were determined by quantitative real time-PCR. (**F**) The protein levels of SOX2 and OCT4 in both AP and AR cells were detected by Western blot analysis. The bars indicate the means ± SD. * *p* < 0.05, ** *p* < 0.005, and *** *p* < 0.001 compared to the AP cells.

Radioresistant A549 cells were generated by exposing the cells to fractionated irradiation at a cumulative dose of 52 Gy (4 Gy × 13 times). The surviving A549 cells after fractionated irradiation were designated as AR cells, while parental A549 cells without irradiation were designated as AP cells. To assess whether AR cells are more radioresistant than AP cells, both AP and AR cells were irradiated once with 2, 4, 6, or 8 Gy of γ-rays. One day after single irradiation, the cell viability was measured by a CCK-8 assay. Consequently, the cell viability of AR cells was significantly higher than that of AP cells (Figure 1A). The viability of AR cells increased significantly at 2 Gy and 4 Gy, suggesting that this result is more resistant to radiation than AP cells. Moreover, the colony-forming ability of AR cells was increased compared to AP cells at 2 Gy and 4 Gy, but not at 6 Gy (Figure 1B). 

Meanwhile, cancer stem cells in heterogenous cancer cells are well known for their resistance to IR. Therefore, we next investigated the expression of cancer stemness-related markers in AR and AP cells. Shown in Figure 1C, the protein expressions of CSC markers such as CD44, ALDH1A1, and CD133 were increased in AR cells and their high expression was maintained even 20 days after final irradiation. The high expression level of CD44 on the AR cell surface was observed, as seen by the analysis of flow cytometry (Figure 1D). AR cells also showed upregulated mRNA and protein levels of OCT4 and SOX2 (Figure 1E,F) and mRNA levels of LIF, SNAIL, MMP2, and MMP7 (Figure 1E) when compared to AP cells. These results suggested that surviving A549 cells after repeated irradiation had the typical characteristics of cancer stem cells. 

### 2.2. Fractionated Ionizing Radiation Enhances Migration and Invasion of A549 Cells

**Figure 2:**
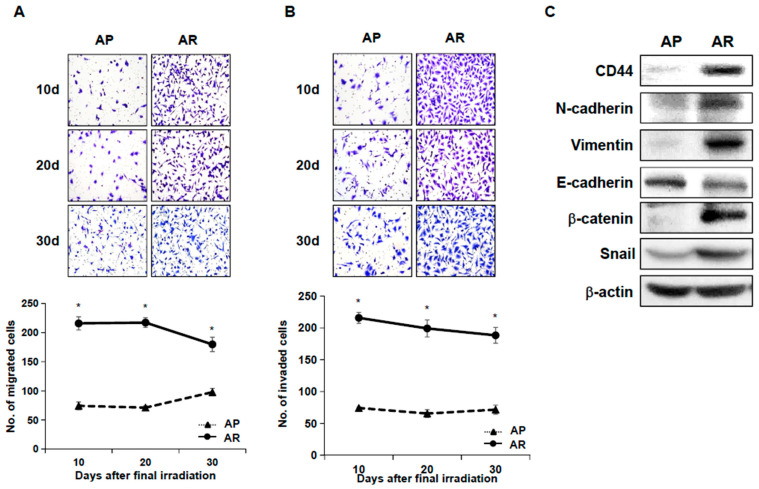
Fractionated ionizing radiation enhances migration and invasion of A549 cells. Both AP cells and AR cells were incubated in transwell for migration assay (**A**) or in Matrigel for invasion assay (**B**) at the days indicated after final irradiation. The photographs are representative fields of migrated or invaded cells on the membrane. The graph indicates the average of migrated or invaded cell numbers from three independent experiments. The bars indicate the means ± SD. * *p* < 0.001 compared to the AP cells. (**C**) Both AP cells and AR cells were lysed, and the lysates were subjected to Western blot analysis.

Cancer stem cells show typical characteristics of metastatic properties. To investigate the metastatic properties of surviving A549 cells after fractionated irradiation, migration and invasion assays were performed in AP cells and AR cells. AR cells exhibited enhanced migration ability, compared to AP cells (Figure 2A). Next, AP cells or AR cells were applied to invasion chambers and the number of adhesive cells was counted to examine cell invasion ability. Shown in Figure 2B, AR cells significantly enhanced invasive ability compared with AP cells. The increased migratory and invasive abilities of AR cells were maintained for up to 30 days after final irradiation, suggesting that surviving A549 cells following repeated irradiation acquired enhanced cell motility. EMT has been shown to endow cancer cells with migratory and invasive properties enabling the initiation of metastasis, which may be closely linked to cancer stem cells [26,29,45]. Therefore, we examined the expression of EMT markers by Western blot analysis. Seen in AR cells, the expression of EMT positive markers (N-cadherin, Vimentin) and transcription factors regulating EMT (Snail, β-catenin) were increased, while the expression of E-cadherin, an EMT negative marker, was down-regulated compared to AP cells (Figure 2C). 

### 2.3. Fractionated Ionizing Radiation Induces Radioresistance through TGF-β-Smad2/3-DLX2 Signaling

**Figure 3:**
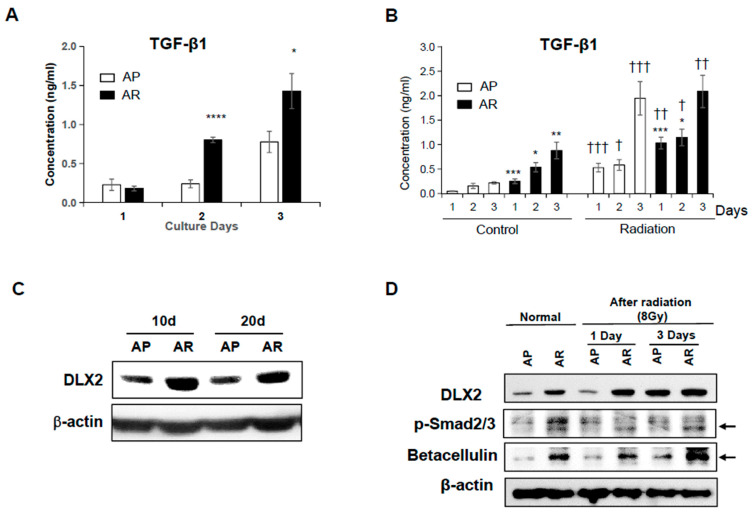
Fractionated ionizing radiation induces TGF-β-Smad2/3-DLX2 signaling. (**A**) AP cells and AR cells were cultured for 1, 2, and 3 days and then the levels of immunoreactive TGF-β1 in the culture medium were quantified by ELISA. (**B**) Both AP cells and AR cells were irradiated at 8 Gy, and the TGF-β1 levels in the culture media after 1, 2, and 3 days were measured by ELISA. (**C**) The expression of DLX2 was determined by Western blot analysis from the lysates of AP cells and AR cells. Similar results were obtained from three independent experiments. (**D**) AP cells and AR cells were irradiated at 8 Gy and cell lysates were obtained at 1 and 3 days after irradiation and subjected to western blot analysis. Similar results were obtained from two independent experiments. The bars indicate the means ± SD. * *p* < 0.05, ** *p* < 0.01, *** *p* < 0.005, **** *p* < 0.001 compared to the AP cells. † *p* < 0.05, †† *p* < 0.005, ††† *p* < 0.001 compared to the control groups.

To determine TGF-β1 secretion by AP and AR cells, the TGF-β1 level in the culture medium was measured using ELISA. The levels of TGF-β1 in the media from both AP cells and AR cells increased time-dependently. Interestingly, AR cells secreted significantly larger amounts of TGF-β1 into the culture medium than AP cells (Figure 3A). Regarding both AP cells and AR cells, single irradiation (8 Gy) significantly increased TGF-β1 secretion time-dependently (Figure 3B). TGF-β1 levels were higher for AR cells than for AP cells at 1 and 2 days after irradiation, but AR cells and AP cells showed similar TGF-β1 levels at 3 days after irradiation.

Given the fact that TGF-β induces cell motility and the invasiveness of cancer cells through EMT [34], the high secretion of TGF-β in AR cells is an expectable result. Conversely, TGF-β also is known to play as a tumor suppressor by promoting cell-cycle arrest and apoptosis [34,35,46]. Recently, Yilmaz and colleagues demonstrated that DLX2 attenuates growth-suppressive TGF-β signaling and promotes cell survival by inducing the expression of the EGFR-ligand betacellulin [42]. Moreover, we previously reported that a single exposure to IR induced DLX2 expression, resulting in acquiring the EMT properties in A549 cells [38]. Therefore, we investigated the involvement of DLX2 and betacellulin in the radioresistance and acquisition of EMT mediated by TGF-β1 in AR cells. Shown in Figure 3C, the expression of DLX2 increased in AR cells, and its high expression maintained for up to 20 days after final irradiation. Single irradiation of AP and AR cells led to the increased expression of DLX2, and a more prominent increase of DLX2 expression was observed in AP cells similar to AR cells at 3 days following irradiation (Figure 3D). 

Meanwhile, TGF-β signaling is mediated by canonical, Smad-dependent signaling [47]. The expression levels of phosphorylated Smad2/3 were enhanced in AR cells compared with AP cells (Figure 3D). However, the irradiation at 8 Gy did not affect the levels of physphorylated Smad2/3 in both AR and AP cells. Betacellulin, a ligand of EGFR and target of Smad-dependent DLX2 signaling, markedly increased in AR cells compared to AP cells. Betacellulin increased in both AP cells and AR cells irradiated at 8 Gy compared with non-irradiated cells (Figure 3D). These results support the hypothesis that acquirement of radioresistance in AR cells relies on DLX2 expression via TGF-β-Smad2/3 signaling.

### 2.4. Knockdown of DLX2 Attenuates the Characteristic of Cancer Stemness and Cancer Cell Proliferation In Vitro

**Figure 4:**
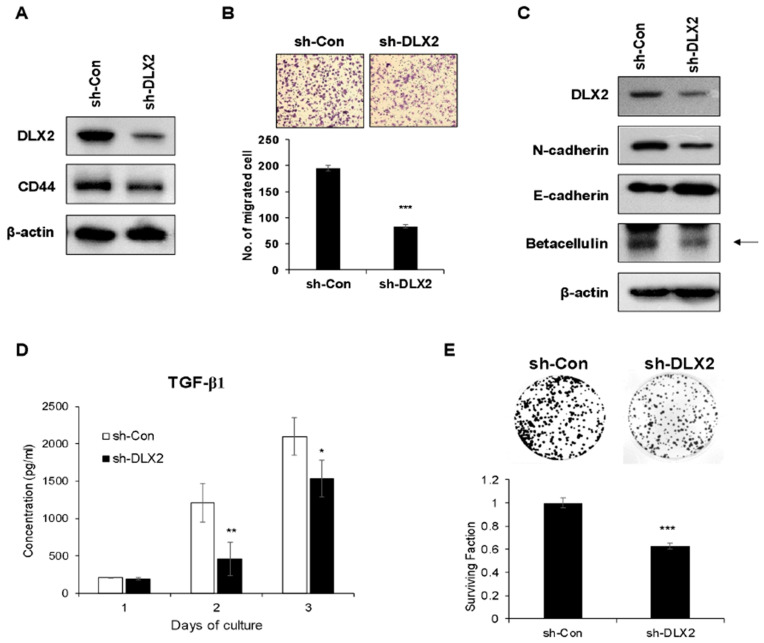
Knockdown of DLX2 suppresses cancer stemness marker CD44 expression and cell migration in A549 cells. (**A**) CD44 protein levels in A549 cells stably expressing shRNA against DLX2 or control shRNA was detected by Western blot analysis. (**B**) The migratory ability of A549 cells stably expressing shRNA against DLX2 or control shRNA were assayed by a transwell migration assay. The photographs are representative fields of migrated cells on the membrane. The graph indicates the average of migrated cell numbers from two independent experiments. (**C**) Lysates of A549 cells stably expressing shRNA against DLX2 or control shRNA were analyzed by Western blot analysis with antibodies against N-cadherin, E-cadherin, and betacellulin. Similar results were obtained from two independent experiments. (**D**) The levels of immunoreactive TGF-β1 in the culture medium of A549 cells stably expressing shRNA against DLX2 or control shRNA were quantified by ELISA. (**E**) A549 cells stably expressing shRNA against DLX2 or control shRNA were incubated for 7 days, and colonies containing 50 cells or more were counted as clonogenic cells. Data were expressed as the surviving fraction. The values represent the means of six replicates from two independent experiments. The bars indicate the means ± SD. * *p* < 0.05, ** *p* < 0.01, *** *p* < 0.001 compared to A549 cells stably expressing control shRNA.

To analyze DLX2 functions on cancer stem-like properties such as radioresistance and metastasis, DLX2 expression in A549 cancer cells was suppressed by stable expression of shRNA against DLX2 (sh-DLX2 A549) and compared to cells transfected with control shRNA (sh-Con A549). Shown in Figure 4A, the suppression of DLX2 was confirmed in sh-DLX2 A549 cells. Moreover, CD44 expression also decreased in sh-DLX2 A549 cells compared to sh-Con A549 cells (Figure 4A). We next compared the migrated cell numbers and the expression levels of EMT-related proteins between sh-Con A549 cells and sh-DLX2 A549 cells. Migration ability was decreased significantly by the knockdown of DLX2 (Figure 4B). Moreover, DLX2 knockdown prevented EMT, as demonstrated by a markedly reduced level of N-cadherin and an increased level of E-cadherin (Figure 4C). These results demonstrated that suppression of DLX2 significantly reduced the motility and EMT of A549 cancer cells.

Since DLX2-mediated acquisition of CSC properties and radioresistance appears to rely on the TGF-β signaling and betacellulin expression, we analyzed the levels of TGF-β1 secreted in the culture medium and the expression levels of betacellulin in sh-Con A549 cells and sh-DLX2 A549 cells. The levels of TGF-β1 in the culture medium were significantly lower in sh-DLX2 A549 cells compared to sh-Con A549 cells (Figure 4D). Also, the betacellulin level was decreased by DLX2 knockdown (Figure 4C). Furthermore, the colony-forming ability of sh-DLX2 A549 cells was significantly decreased, suggesting that reduction of betacellulin expression via DLX2 knockdown lowers proliferation of A549 cells (Figure 4E). 

### 2.5. Knockdown of DLX2 Results in a Reduction of Tumor Growth

**Figure 5:**
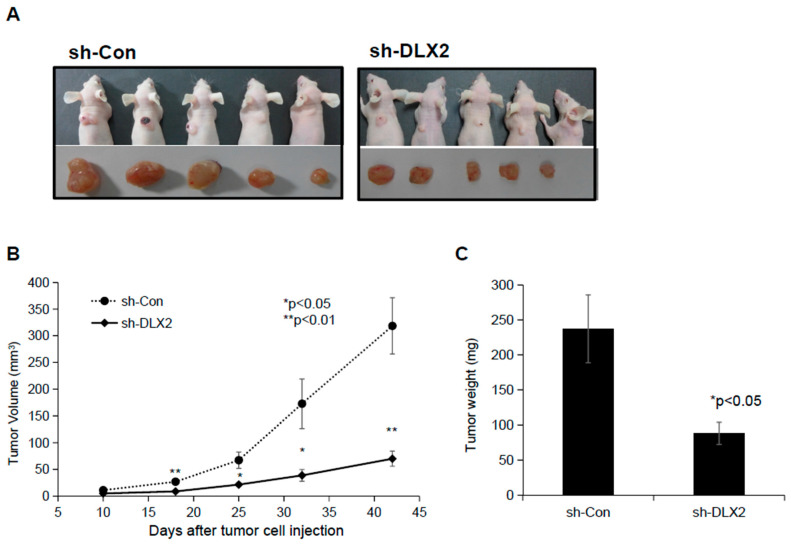
Knockdown of DLX2 in A549 cells decreases the tumor growth in the xenograft experiment. A549 cells stably expressing shRNA against DLX2 or control shRNA were injected into the back of BALB/c nude mice. (**A**) Forty-two days after tumor cell injection, all mice were sacrificed, and tumor tissues were collected. (**B**) Tumor volumes were determined at the indicated days after tumor cell injection. The bars indicate the means ± SE (*n* = 5 per each group). (**C**) The weights of the tumors collected at 42 days after cell injection were measured. The values represent the means ± SD (*n* = 5 for each group). * *p* < 0.05, ** *p* < 0.01 compared to A549 cells stably expressing control shRNA.

To investigate whether suppression of DLX2 impaired the ability of A549 cells to form tumors, athymic BALB/c nude mice were injected subcutaneously with sh-Con or sh-DLX2 A549 cells, and the tumor volume and tumor weight were measured. Shown in Figure 5B, on day 42, the tumor volume in mice injected with sh-Con A549 cells increased to 318.5 ± 129.3 mm^3^, whereas it was only 70.1 ± 31.8 mm^3^ in mice inoculated with sh-DXL2 A549 cells. These differences were visible from day 18. Similar results were observed in the tumor weight measured on day 42 (Figure 5C). These results indicated that DLX2 expression is required for tumor growth in vivo.

## 3. Discussion

The incidence and mortality of lung cancer ranks as one of the highest among all cancers worldwide [48]. Radiotherapy is a double-edged sword: although IR is used as a standard treatment for lung cancer patients, its therapeutic efficacy is limited due to the tumor recurrence and metastasis and the induction of damages in normal tissues. Previous researchers have observed that IR promotes stemness properties in heterogeneous cancer cells [23,49]. CSCs are believed to play a key role in cancer metastasis, cancer recurrence, and radiation and anticancer drug resistance [5,6,7,8,9]. During this present study, we demonstrated that repeated irradiation of A549 NSCLC cells led to acquiring radioresistance and inducing the expression of CSC-related markers such as CD44, OCT4, SOX2, LIF, Snail, MMP2, and MMP7. Gao and colleagues recently found the effective dose of a single exposure of IR for significantly enhancing the size of the stem cell pool [49]; their results presented the requirement of 10 Gy or more to enrich the CSCs in the heterogenous breast cancer cell line (MCF-10A and MCF-7). Conversely, exposure of cells to radiation, especially at low doses, can mediate genomic instability, adaptive responses, and epigenetic changes, leading to induction of spherogenesis in non-stem cancer cells [23]. Martines-Neves and colleagues recently suggested that the enrichment of stem-like cancer cells after IR exposure can occur in two mechanisms: IR-induced stemness or CSC selection [27]. They described that these mechanisms work depending on the temporal window, time of exposure, dosage, and concentration, which can lead to induction of a stem cell phenotype from heterogenous cancer cell lines and selective enrichment of CSCs. We previously reported that single exposure of IR promoted the expression of CSC markers in human lung and breast cancer cell lines [38]. Here we argue one more thing: the enrichment of CSCs from a heterogenous cancer cell line is more effectively achieved by repetitive irradiations at low doses than by a single radiation at a high dose.

Generally, EMT is a multistep biological process by which epithelial cells undergo morphological changes characterized by a transition from the epithelial cobblestone phenotype to the elongated fibroblastic phenotype. EMT can be induced by IR [38,50,51] and is closely related to CSCs [25,26]. EMT has been shown to play important roles in the acquisition of stemness in cancer cells, and leads to the migratory and invasive properties, enabling the initiation of metastasis [26,29,45]. Recently, we and other researchers have reported that surviving cancer cells following single irradiation showed an EMT and cancer stemness phenotype [25,38,49]. During the present study, surviving A549 cells following repeated irradiation with γ-rays remarkably increased migration and invasion and, also, showed the downregulation of epithelial molecular markers such as E-cadherin and the upregulation of mesenchymal molecular markers such as vimentin, N-cadherin, β-catenin, and Snail. These properties of surviving cancer cells were maintained at least 30 days after the final irradiation.

Although EMT is activated by oncogenic pathways such as Src, Ras, Wnt, and Notch, TGF-β is the most powerful inducer for IR-induced EMT [50]. During tumor radiotherapy, TGF-β is produced locally not only from cancer cells but also from surrounding normal cells, and probably is one of the most important players in the IR response [52,53]. Our report in this study shows that surviving radioresistant A549 (AR) cells following repeated irradiation secreted more TGF-β1 than normal parental A549 (AP) cells. We postulated that AR cells acquired CSC and EMT properties through TGF-β signaling. During tumor progression, the TGF-β signaling pathway exerts a dual function: TGF-β exerts tumor suppressive effects by inducing cell cycle arrest and apoptosis, while also promoting tumor cell invasion and metastasis by inducing immune regulation, EMT, and angiogenesis [35,36,46]. It still is poorly understood how cancer cells evade the TGF-β-mediated tumor-suppressive barrier. TGF-β-mediated expression of genes encoding pro-apoptotic factors such as the cell cycle inhibitors p15^INK4B^ and p21^CIP1^ [47] is mediated by canonical Smad-dependent signaling, while the acceleration of tumor progression, survival, and EMT is activated by non-canonical TGF-β signaling such as MAPK and PI3K pathways [54]. We previously reported the increased expression of DLX2 in human lung and breast cancer cell lines exposed to single IR at 8 Gy and these cells showed TGF-β-mediated EMT by Smad2/3 signaling [38].

DLX2 is a homeobox transcription factor and its association with tumor progression and metastasis has been reported [41,42,43]. Recently, Yilmaz and colleagues demonstrated that TGF-β-induced DLX2 represses the transcription of TGFβRII by directly binding to its promoter for a negative feedback loop, and also induces mitogenic epidermal growth factor receptor (EGFR) signaling by directly inducing the expression of the EGFR-ligand betacellulin [42]. It also was reported that DLX2 is involved in TGF-β-induced/Wnt-induced EMT via Snail activation [44]. These findings suggested that DLX2 plays an important role in tumor progression and metastasis in TGF-β-exposed cancer cells. During this study, we demonstrated that overexpression of transcription factor DLX2, activation of Smad2/3 signaling, and increased expression of betacellulin were observed in A549 cancer cells surviving the fractionated irradiation. Interestingly, the loss of DLX2 in A549 cells dramatically reduced the CSCs marker (CD44), EMT marker (N-cadherin), and the property of cell migration as well as TGF-β1 and betacellulin expression. Also, DLX2-knockdown A549 cells showed a markedly decreased survival and tumorigenic capability, consistent with the previous finding that the loss of DLX2 function in mouse retinal cells results in increased apoptosis [55]. Although the increased TGF-β are in an active form or in an inactive form with attached LAP is very important, this investigation was unfortunately not included in our current study, but I think it should be investigated in our future study.

Collectively, we demonstrated that NSCLC cells surviving after fractionated irradiation with γ-rays acquired a stemness state characterized by increasing stemness gene expression. During previous studies, IR-induced CSCs is associated with activation of self-renewal signaling pathways such as Wnt/β-catenin, Notch, and Hedgehog [56,57]. Although further investigations are necessary to clarify the role of DLX2 in IR-induced CSCs, here we provide novel and attractive insights into the regulation and function of DLX2 on cancer stemness, radioresistance, tumor survival/proliferation and EMT in surviving NSCLC cells following repeated exposure with γ-rays.

## 4. Materials and Methods

### 4.1. Cell Culture and Irradiation

A human alveolar type II epithelial carcinoma cell line (A549, NSCLC) was purchased from the Korean Cell Line Bank (Seoul, Korea). Cells were cultured in Dulbecco’s modified Eagle’s medium (DMEM; Life Technologies, Inc., Carlsbad, CA, USA) supplemented with 10% heat-inactivated fetal bovine serum (FBS; Gibco) and antibiotics (100 U/mL penicillin and 0.1 mg/mL streptomycin) at 37 °C in a standard humidified 5% CO_2_ atmosphere. Irradiation of A549 cells was conducted at room temperature by exposure to a ^137^Cs γ-ray with a Gammacell 40 Exactor (Nordion International Inc., Ottawa, ON, Canada). A549 cells were irradiated at 4 Gy once every five days to a cumulation dose of 52 Gy (4 Gy × 13 times) to make cell lines resistant against IR. Following these irradiations, surviving A549 cells were designated as AR cells, while control A549 cells cultured for the same period without irradiation were designated as AP cells. AR cells were stored in liquid nitrogen three to four days after the last irradiation and used in experiments. 

### 4.2. Cell Counting Kit-8 (CCK-8) Assay for Cell Viability

To measure the cell viability of AP cells and AR cells against irradiation, these cells were exposed to various doses of γ-rays and inoculated into five wells of the 96-well plates (1 × 10^4^ per well). Twenty-four hours after culture, 5 μL of CCK-8 (Dojindo; Kumamoto, Japan) reagent was added to the cells for 3 h and the absorbance was measured at 450 nm using a microplate reader (Molecular Devices, Sunnyvale, CA, USA). 

### 4.3. Colony Forming Assay

To measure the radioresistance, AP cells and AR cells were exposed to various doses of γ-rays and plated into six wells at 500 cells per well in 6-well plates. The medium of all cultures was refreshed every 3 days. Seven days later the colonies were fixed with 60% methanol and stained with 0.5% crystal violet. Colonies containing 50 cells or more were counted as clonogenic cells. Surviving fraction (SF) and plating efficacy (PE) were calculated using the equation; SF = PE of test group/PE of control group, and PE = (No. of colony/No. of seeding cells) × 100. The surviving fraction value was reported as the mean of eighteen wells from three independent experiments.

### 4.4. Cell Migration Assay

To measure cell migration, cells were seeded in a trans-well (Corning Incorporated, New York, NY, USA) at a density of 2.5 × 10^4^ cells/well in 200 μL of serum-free medium. Seven hours later the medium was removed and cells were then stained with 0.5% crystal violet for 15 min after fixing with 100% methanol. The membrane was cut away from each chamber and migrated cells on the lower surface of the filter were counted per filter in a random microscopic field at a 200-fold magnification with Leica DM LM microscope (Leica, Heidelberg, Germany). The value was reported as the mean of three independent experiments.

### 4.5. Cell Invasion Assay

The ability of AP cells and AR cells to pass through matrigel-coated filters (pore size, 8 μm) was measured in a Boyden chamber invasion assay. Cell invasion assays were performed using a matrigel invasion assay kit (BD Biosciences, Bedford, MA, USA) according to the manufacturer’s instructions. Briefly, AP cells or AR cells were seeded in the upper chamber at a density of 2.5–5 × 10^4^ cells/well in 500 μL of serum-free medium and incubated for 48 h. Invaded cells on the lower surface of the filter were fixed with 100% methanol and stained with 0.5% crystal violet for 15 min. The membrane was cut away from each chamber and invaded cells were counted per filter in a random microscopic field at a 100-fold magnification. The value was reported as the mean of three independent experiments. 

### 4.6. Western Blot Analysis

Total cell lysates were prepared using RIPA lysis buffer (Thermo Scientific, Rockford, IL, USA) containing 10 mM phenylmethanesulphonyl fluoride (PMSF), 10 mM sodium fluoride (NaF), 1 mM sodium orthovanadate (Na_3_VO_4_) and a complete protease inhibitor cocktail (Sigma-Aldrich, St. Louis, MO, USA) for 30 min on ice. The protein concentrations of the cell extracts were measured using the BCA (bicinchoninic acid) method (Pierce, Rockford, IL, USA) with bovine serum albumin (BSA) as the standard. Cell lysates were boiled in a Laemmli sample buffer containing 2-mercaptoethanol (Bio-Rad Laboratories, Inc., Hercules, CA, USA). Equal amounts (10~20 μg) of protein were separated on SDS-polyacrylamide gel and then transferred onto a Hybond^TM^-P membrane (Amersham, Freiburg, Germany). After blocking in a buffer (50 g/L skimmed milk or BSA in tris-buffered saline containing 0.1% (*v*/*v*) tween-20 (TBST)), the membranes were incubated overnight at 4 °C with primary antibodies. The primary antibodies were as follows: antibodies against DLX2, Smad2/3, CD44, β-catenin, N-cadherin, and E-cadherin were purchased from Thermo Scientific (Rockford, IL, USA), anti-Snail, anti-vimentin, and β-actin were purchased from Cell Signaling (Banvers, MA, USA), and anti-phospho-Smad2/3 antibody was purchased from Kerafast, Inc. (Boston, MA, USA). After washing with TBST, the membranes were incubated with anti-goat IgG antibody, anti-rabbit IgG antibody, or anti-mouse IgG conjugated with horseradish peroxidase (HRP) (Cell Signaling, Banvers, MA, USA) for 1 h, and developed using ECL Western blot detection reagents (Millipore, Billerica, MA, USA). 

### 4.7. Flow Cytometry Analysis

To analyze CSC properties in surviving A549 cells after repeated irradiation, cells were stained with monoclonal antibodies against CD44 (Thermo Scientific, Rockford, IL, USA) for 30 min at room temperature, and then analyzed using a FACSCalibur flow cytometer (BD Biosciences, San Jose, CA, USA). A fluorescence histogram of at least 20,000 counts was obtained for each sample. The FITC-labeled CD44 monoclonal antibody and isotype IgG2b antibody were purchased from Abcam (Cambridge, UK). 

### 4.8. RNA Extraction and Quantitative Real-Time PCR

Total RNA was isolated from cells using TRIzol Reagent (Thermo Fisher Sicentific, Wiltham, MA, USA) according to the manufacturer’s protocol. Reverse transcription from three micrograms of total RNA was implemented using TOPscript RT Dry MIX containing reverse transcriptase, RT buffer, dNTP mixture Oligo dT (Enzynomics, Seoul, Korea). Quantitative real-time PCR was performed by a StepOne Real-Time PCR (Thermo Fisher Sicentific, Waltham, MA, USA) with SYBR Green reagent. Primers were designed using Primer-BLAST (http://www.ncbi.nlm.nih.gov/tools/primer-blast/, accessed on 16 January 2021) and the sequences are presented in Table 1. The PCR amplifications were carried out in a 20 μL reaction solution and the reaction condition was as follows: 15 min of initial denaturation at 95 °C, 40 cycles of 10 s at 95 °C, 15 s at 60 °C, and 20 s at 72 °C. The comparative C_t_ method was used and the relative mRNA expression level was calculated based on normalization to β-actin. All experiments were repeated three times independently.

### 4.9. Measurement of TGF-β1 in Cell Culture Supernatant

The level of TGF-β1 in the culture supernatant was measured by a BD OptEIA^TM^ ELISA set (BD PharMingen, San Diego, CA, USA) according to the manufacturer’s instructions. The absorbance at 450 nm was measured using a microplate reader (Molecular Devices, Sunnyvale, CA, USA). The TGF-β1 protein level was determined as the mean of the values from three independent experiments.

### 4.10. Establishment of shRNA-Based DLX2 Knockdown A549 Cells

MISSION shRNA vector systems containing a DLX2 shRNA sequence (pLKO.1-sh-DLX2) or a control shRNA sequence (pLKO.1-sh-control) were purchased from Sigma-Aldrich (St. Louis, MO, USA) and were used to establish the stable DLX2 knockdown cells or control cells. The hairpin sequences of pLKO.1-sh-DLX2 and pLKO.1-sh-control were 5′-CCGGGCCTGAAATTCGGATAGTGAACDCGAGTTCACTATCCGAATTTCAGGC- TTTTT-3′ and 5′-CCGGTACAACAGCCACAACGTCTATCTCGAGATAGACGTTGTG-GCTGTTGTATTTTT-3′, respectively. A549 cells were plated at 1.3 × 10^6^ cells in a 100 mm culture dish. After 24 h, cells were transfected with 10 μg of pLKO.1-sh-control or pLKO.1-sh-DLX2 using a jetPRIME transfection reagent (Polyplus transfection, Illkirch, France) per the manufacturer’s instructions. After 48 h, cells were sub-cultured in the medium containing 1 μg/mL puromycin. The culture medium containing 1 μg/mL puromycin was refreshed every 2–3 days. After 11 days, the surviving cells were detached with 2.5% Trypsin/EDTA and were transferred to 96-well plates so each well contained one cell on average. After culturing for an additional 2–3 weeks while refreshing the medium containing puromycin every 2–3 days, the cell clones that formed one colony in one well were transferred to a 60 mm culture dish. The clones were checked for the chromosomal integration of pLKO.1-sh-DLX2 by real-time PCR (forward primer: GACGAGGTACCGGCTCACTA, reverse primer: TGTGGATGAATACTGCCATTTGTC). DLX2 protein levels in the clones were examined by Western blot. An A549 clone with chromosomal integration of pLKO.1-sh-DLX2 and a low DLX2 protein level was selected and used for further in vitro experiments and the xenograft tumor growth assay.

### 4.11. Xenograft Tumor Growth Assay

Six-week-old female athymic BALB/c female nude mice were purchased from OrientBio Inc. (Charles River Technology; Seongnam, Republic of Korea). The sh-control or sh-DLX2 A549 cells were subcutaneously inoculated (4 × 10^5^ cells/mouse) into eight-week-old nude mice (*n* = 5/group). Tumor volume was measured by a caliper every 7~10 days from 10 days to 42 days after inoculation, and their volumes were calculated using the following equation: V(mm^3^) = length(mm) × width(mm)^2^/2. Six weeks after inoculation, tumor xenografts were excised and their weight was measured. The animal experiment was conducted according to the guidelines for the use and care of laboratory animals of Ministry of Health and Welfare, Republic of Korea. The experimental protocol was approved (KAERI-IACUC-2018-014) by the Institutional Animal Care and Use Committee of KAERI and the criteria for euthanasia to minimize suffering was included therein.

### 4.12. Statistical Analysis

All experiments were performed at least three times independently. All data were expressed as mean ± S.D. except Figure 5B (mean ± S.E.) and the statistically significant difference was analyzed using the two-tailed Student’s *t*-test.

## Figures and Tables

**Table 1:** Primer sequence for Quantitative real-time PCR.

Primer	Sequences
OCT4	
Forward	5′-AGCAAAACCCGGAGGAGT-3′
Reverse	5′-CCACATCGGCCTGTGTATATC-3′
SOX2	
Forward	5′-GTGAGCGCCCTGCAGTACAA-3′
Reverse	5′-GCGAGTAGGACATGTGTAGGTG-3′
LIF	
Forward	5′-GTTCCCCAACAACCTGGACA-3′
Reverse	5′-ACGACTATGCGGTACAGCTC-3′
SNAIL	
Forward	5′-TTTCCTCGTCAGGAAGCCCTC-3′
Reverse	5′-TGCTGGAAGGTAAAACTCTGGATTAG-3′
MMP2	
Forward	5′-GGAAAGCCAGGATCCATTTT-3′
Reverse	5′-ATGCCGCCTTTAACTGGAG-3′
MMP7	
Forward	5′-GTCACTTCTTCGGTTGTAGGGA-3′
Reverse	5′-TCAGAGGAATGTCCCATACCCA-3′
β-actin	
Forward	5′-GACCTGTACGCCAACACAGT-3′
Reverse	5′-CCAGGGCAGTGATCTCCTTC-3′

## Data Availability

The data that support the findings in this study are available from the corresponding authors upon reasonable request.
